# Comparative Metagenomics Reveals Host Specific Metavirulomes and Horizontal Gene Transfer Elements in the Chicken Cecum Microbiome

**DOI:** 10.1371/journal.pone.0002945

**Published:** 2008-08-13

**Authors:** Ani Qu, Jennifer M. Brulc, Melissa K. Wilson, Bibiana F. Law, James R. Theoret, Lynn A. Joens, Michael E. Konkel, Florent Angly, Elizabeth A. Dinsdale, Robert A. Edwards, Karen E. Nelson, Bryan A. White

**Affiliations:** 1 Department of Animal Sciences, University of Illinois, Urbana, Illinois, United States of America; 2 Department of Veterinary Science and Microbiology, University of Arizona, Tucson, Arizona, United States of America; 3 School of Molecular Biosciences, Center for Biotechnology, Washington State University, Seattle, Washington, United States of America; 4 Department of Biology, San Diego State University, San Diego, California, United States of America; 5 Department of Computational Science, San Diego State University, San Diego, California, United States of America; 6 School of Biological Sciences, Flinders University, Adelaide, Australia; 7 Center for Microbial Sciences, San Diego State University, San Diego, California, United States of America; 8 Department of Computer Sciences, San Diego State University, San Diego, California, United States of America; 9 The J. Craig Venter Institute, Rockville, Maryland, United States of America; 10 The Institute for Genomic Biology, University of Illinois, Urbana, Illinois, United States of America; Centre for DNA Fingerprinting and Diagnostics, India

## Abstract

**Background:**

The complex microbiome of the ceca of chickens plays an important role in nutrient utilization, growth and well-being of these animals. Since we have a very limited understanding of the capabilities of most species present in the cecum, we investigated the role of the microbiome by comparative analyses of both the microbial community structure and functional gene content using random sample pyrosequencing. The overall goal of this study was to characterize the chicken cecal microbiome using a pathogen-free chicken and one that had been challenged with *Campylobacter jejuni*.

**Methodology/Principal Findings:**

Comparative metagenomic pyrosequencing was used to generate 55,364,266 bases of random sampled pyrosequence data from two chicken cecal samples. SSU rDNA gene tags and environmental gene tags (EGTs) were identified using SEED subsystems-based annotations. The distribution of phylotypes and EGTs detected within each cecal sample were primarily from the *Firmicutes*, *Bacteroidetes* and *Proteobacteria*, consistent with previous SSU rDNA libraries of the chicken cecum. Carbohydrate metabolism and virulence genes are major components of the EGT content of both of these microbiomes. A comparison of the twelve major pathways in the SEED Virulence Subsystem (metavirulome) represented in the chicken cecum, mouse cecum and human fecal microbiomes showed that the metavirulomes differed between these microbiomes and the metavirulomes clustered by host environment. The chicken cecum microbiomes had the broadest range of EGTs within the SEED Conjugative Transposon Subsystem, however the mouse cecum microbiomes showed a greater abundance of EGTs in this subsystem. Gene assemblies (32 contigs) from one microbiome sample were predominately from the *Bacteroidetes*, and seven of these showed sequence similarity to transposases, whereas the remaining sequences were most similar to those from catabolic gene families.

**Conclusion/Significance:**

This analysis has demonstrated that mobile DNA elements are a major functional component of cecal microbiomes, thus contributing to horizontal gene transfer and functional microbiome evolution. Moreover, the metavirulomes of these microbiomes appear to associate by host environment. These data have implications for defining core and variable microbiome content in a host species. Furthermore, this suggests that the evolution of host specific metavirulomes is a contributing factor in disease resistance to zoonotic pathogens.

## Introduction

Microorganisms and their complex microbial communities are responsible for most of the biochemical transformations in the environment. The gastrointestinal tract of animals harbors a large, complex, and dynamic microbial community, and the composition of this community ultimately reflects the co-evolution or selection of microorganisms with their animal host and the diet adopted by the host. As a result of issues that relate to food safety and animal nutrition and health, the structure and function of the gut microbial community has received significant attention from researchers. The majority of these microbial species cannot be cultured under traditional culturing techniques, and therefore, we have a very limited understanding of the capabilities of most species. More recently, with the introduction and growth of molecular tools in microbial ecology, many culture-independent methods have developed to overcome the cultivation biases and allow detailed information on microbial community diversity, structure, and function. The use of the small subunit (SSU) rRNA gene as a phylogenetic marker to study bacterial and archaeal diversity, as well as the composition of various environments and natural communities has resulted in tremendous quantities of information about microbial communities. Nonetheless, these techniques have revealed limited information on the physiological role that is played by individual species that have been identified by SSU rDNA sequencing. SSU rRNA gene surveys continue to expand, and as of 2008 the Ribosomal Database Project (RDP; http://rdp.cme.msu.edu/) holds an estimated 481,650 aligned and annotated 16S rRNA gene sequences, demonstrating the extent of microbial diversity in the environment and hinting at what remains to be discovered.

The sequencing of the genomes from several hundred microbial and numerous eukaryotic species has laid the foundation for generating genomic sequence data from whole environments without first using a culturing step. This approach, also known as “metagenomics” [1], is defined as the genomic analysis of microorganisms by direct extraction and cloning of DNA from an assemblage of microorganisms [1]. Pyrosequencing is the base for a promising new generation sequence technology developed by 454 Life Sciences (http://www.454.com/) [2]–[5] and is now being applied to metagenomics. One approach has been the use of the pyrosequence technology to increase the depth of SSU rDNA surveys by sequencing amplicons from the variable region of the SSU molecule. This has been applied to ocean microbial samples [6], soils [7], and was recently used in a multiplex pyrosequencing study of 286 enviornmental samples that generated 437,544 SSU rDNA tags, nearly as many as have already been generated by Sanger sequencing [8]. The second approach uses random sample pyrosequencing to generate environmental gene tags (EGTs (protein families [9]) from microbiomes. This approach allows one to highlight significant differences in metabolic potential in each environment. This has been applied to environmental biomes [10] as well as the gastrointestinal microbiomes of C57BL/6J mice with or without a mutation in the leptin gene [11], and was recently used to analyze ∼14 million pyrosequences from 45 distinct microbiomes and 42 viromes [12], including the ones analyzed in detail in this study.

While the cloning and sequencing of SSU rDNA, T-RFLP and array based-OFRG has been used to describe the microbial communities of the gastrointestinal tracts of poultry [13]–[27], the functional gene content of these microbiomes has not been studied. One area of interest is the role of commensal gastrointestinal bacteria in *Campylobacter jejuni* colonization of chickens. Investigators have reported that the use of antibiotic growth promotants (AGP), which altered the microbiome, decreased the levels of *Campylobacter* bacteria in chickens reared conventionally versus chickens reared without AGP [28]. Specifically, *Campylobacter* 16S rDNA was detected in the cecal samples of all AGP-free birds at days 14 and 21, but not in chickens reared conventionally. *C. jejuni* colonizes the ceca of chickens at densities of 10^8^ CFU per gram of cecal contents or greater without causing disease [29]–[31]. By two to three weeks of age, most commercially reared poultry are colonized by *C. jejuni* [32]. While day-old chicks can become colonized with *C. jejuni* when experimentally inoculated, natural colonization with *C. jejuni* does not occur until after 2 to 3 weeks of age [30], [33]–[35]. After *C. jejuni* colonizes a few birds in a flock, it rapidly spreads throughout the flock [34], [36]. Once colonized with *C. jejuni*, the bacteria remain present throughout the bird's lifespan [30], [35]. In fact, 50 to 90% of domestic chicken carcasses are contaminated at the time of sale [37], [38]. However, we lack a fundamental understanding of how *C. jejuni* colonization affects the normal cecal community structure and visa versa.

In order to expand on these studies, we applied for the first time to our knowledge, a random sample pyrosequencing approach to the complex microbiome of the cecum of chickens. Our goal was to obtain both phylotype and functional gene content, or the metabolic potential, by a characterization of the microbiome from a pathogen-free chicken and one that had been challenged by a single low-level inoculation with *C. jejuni*. The present study demonstrates that random sample pyrosequencing can provide high fidelity gene assemblies from the microbiome, and revealed that in the chicken cecum, mobile elements are a major functional component of these microbiomes. It also appears that the genes associated with virulence or a “metavirulome” of these microbiomes cluster by host environment. This suggests that the core and variable microbiome content in a host species not only influences the adaptation of mutualistic or commensal microorganisms, but also influences disease resistance to zoonotic pathogens.

## Results

**Figure 1:**
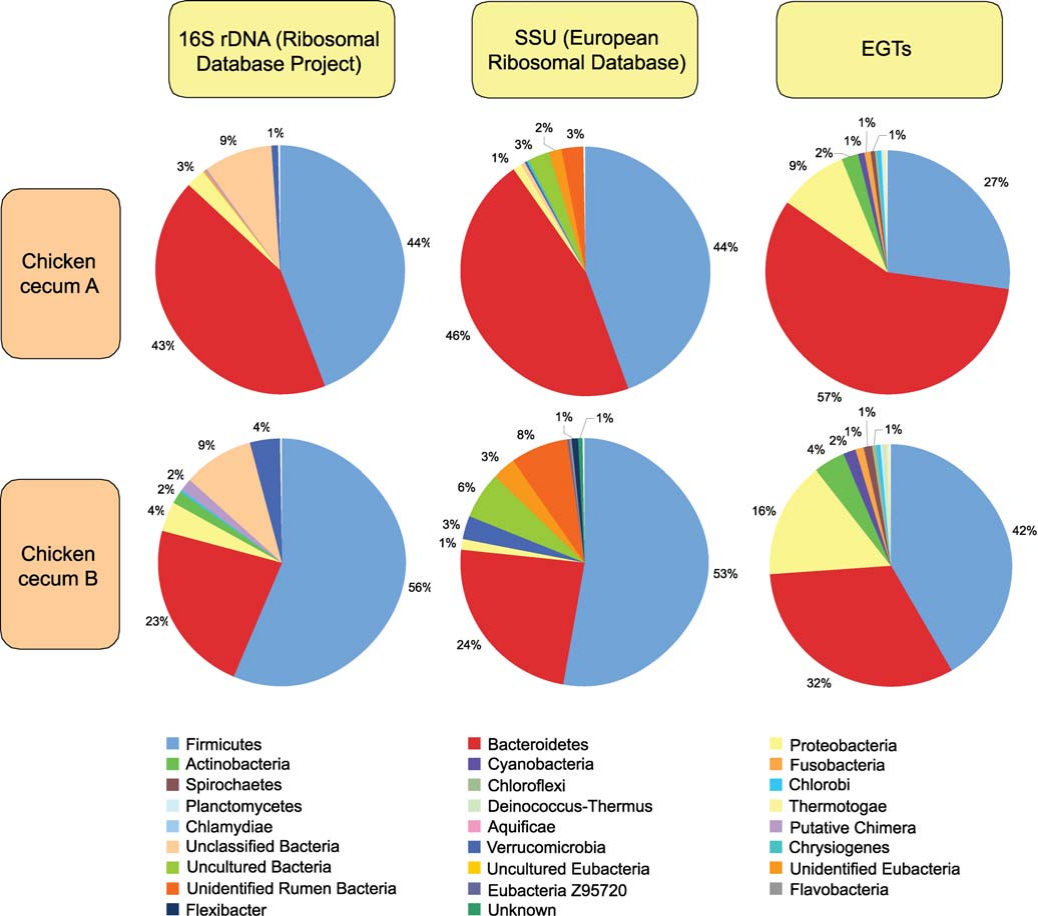
Phylogenetic composition of bacterial phyla from pyrosequence 16S rDNA sequences, and environmental gene tags (EGTs) from two pyrosequenced chicken cecum samples. The percent of sequences in each of the bacterial phyla from the chicken cecum A and B microbiomes is shown. E-value cutoff for SSU rDNA hits for all databases used is 1×10^−5^ with a minimum length of 50 bp. The BLASTX cutoff for EGTs is 1×10^−5^.

**Figure 2:**
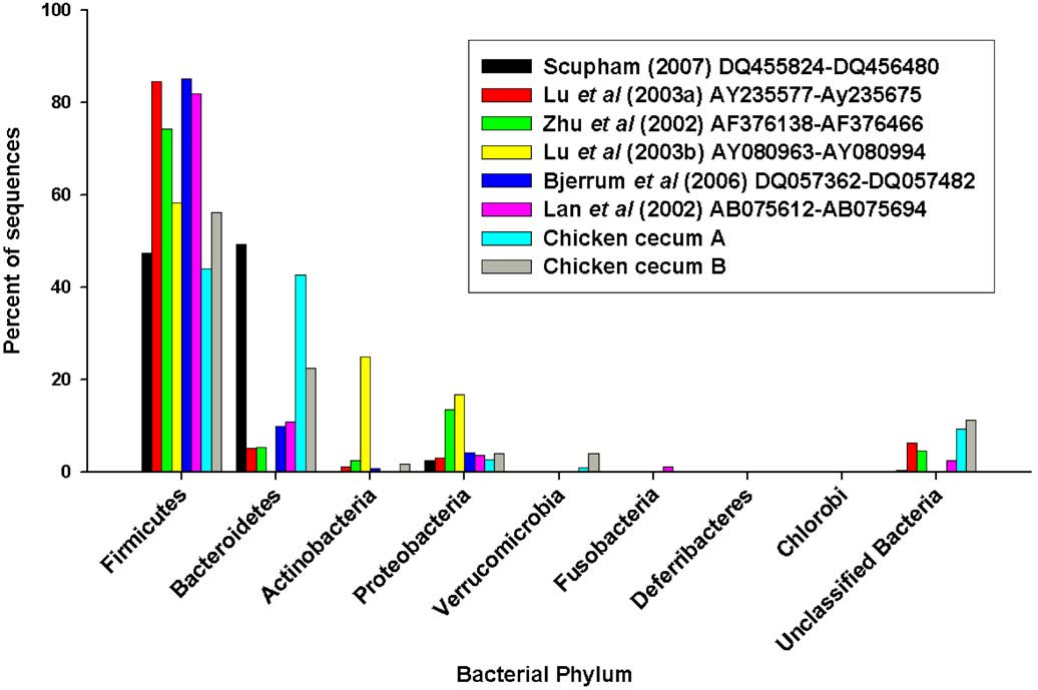
The taxanomic distribution of Bacterial Phylum in eight microbial samples from the cecum of chickens.

In order to better understand the functional gene content and metabolic potential in the chicken cecal microbial community, we undertook a direct large-scale random sample comparative metagenomic strategy using 454 pyrosequencing. The overall goal of this study was to obtain a detailed characterization of the microbiome using a pathogen-free chicken (cecum A), and one that had been challenged by a single low-level inoculation with *C. jejuni* (cecum B), with respect to both phylotype (ribosomal DNA gene tags) and functional content (environmental gene tags; EGTs). Similar relationships (Figure 1 and Table 1) were seen for SSU rDNA hits against the Ribosomal Database Project (Bacterial SSU rDNA), and against European Ribosomal RNA databases (Archaeal and Eukaryotic SSU rDNA). The number of SSU rDNA hits in the chicken cecum metagenomic libraries (Table 1), are consistent with the numbers we found for rumen microbiomes [39]. As expected, the distribution of phylotypes fell predominantly into the *Firmicutes*, *Bacteroidetes* and *Proteobacteria* (Figure 1), regardless of the SSU rDNA database used for the analysis. The taxanomic distribution of the numerically abundant Bacterial Phyla (*Actinobacteria*, *Bacteroides*, *Chlorobi*, *Deferribacteres*, *Firmicutes*, *Fusobacteria*, *Proteobacteria* and *Verrucomicrobia*) were compared between eight poultry cecal SSU rDNA libraries (Wilcoxon exact test P≤0.05) [16], [17], [20], [25]–[27]. The analysis was conducted on the percent of sequences showing similarity to each bacteria phylum, thus normalizing for variance in sequencing depth. There was no difference between any pairing (P>0.05). While there was no difference between samples, the percent of sequences showing similarity in each bacterial group differed (Figure 2). *Firmicutes* were the dominant taxa associated with all chicken ceca. *Bacteriodes* were highly represented in the Chicken cecum A, Chicken cecum B and samples from turkey poult ceca [20]. A high abundance of Actinobacteria was found in the broiler chicken samples [25]. All other taxa were found in low abundance. We only detected one *Campylobacter* SSU rDNA sequence and this was in the cecum B microbiome, from the chicken challenged with *C. jejuni*. No Archaeal and few Eucarya SSU rDNA (∼1%) or mitochondria phylotypes (48 and 19 respectively) were identified in our microbiomes, with the majority most similar to the *Chordata* (i.e., host).

**Table 1:** Summary of pyrosequence data from different chicken cecum samples.

	Chick cecum A	Chick cecum B
Number of sequences	294,682	237,940
Total length of sequences	30,657,259	24,707,007
Ave length of sequences (bp)	104	104
Total coding sequences (EGTs) (% of total sequences)	117,231 (0.38%)	76,424 (0.31%)
Archaea EGTs (% of total EGTs)	951 (0.81%)	847 (1.11%)
Bacteria EGTs (% of total EGTs)	114,074 (97.3%)	74,480 (97.5%)
Broad host range plasmids (% of total EGTs)	1 (0.001%)	2 (0.003%)
Eukarya EGTs (% of total EGTs)	2061 (1.76%)	968 (1.27%)
Plasmids (% of total EGTs)	2 (0.002%)	8 (0.01%)
Virus EGTs (% of total EGTs)	142 (0.12%)	119 (0.16%)
Number of SSU rDNA Hits:		
Ribosome Database Project (% of total sequences)	489 (0.002%)	416 (0.002%)
European Ribosomal RNA Database (% of total sequences)	510 (0.002%)	401 (0.002%)

The BLASTX cutoff for environmental gene tags (EGTs) is 1×10^−5^. E-value cutoff for SSU rDNA hits for all databases used is 1×10^−5^ with a minimum length of 50 bp.

Further insight into the diversity within the two chicken cecum metagenomic samples was obtained by comparing the number of SSU rDNA sequences and EGTs (E value<1×10^−5^) in different bacterial phyla (Figure 1). Sequence length is one of the primary factors in assessing similarity between sequences, and BLAST E values are dependent on both the length of the query sequence and the length of the database to which they are being compared [40]. Although this will affect the number of significant sequences found in the searches by a factor of two or more [41], pyrosequencing yielded orders of magnitude more sequence per dollar than comparable Sanger sequencing, more than compensating for these missing sequences. The sequences missed in our searches are expected to be randomly distributed, and therefore are not expected to skew the comparative analysis. Finally, while classifying EGTs from short pyrosequencing reads has been challenging, a recent report demonstrates that EGTs as short as 27 amino acids can accurately be classified with an average specificity ranging from 97% for Superkingdom to 93% for Order [42].

**Figure 3:**
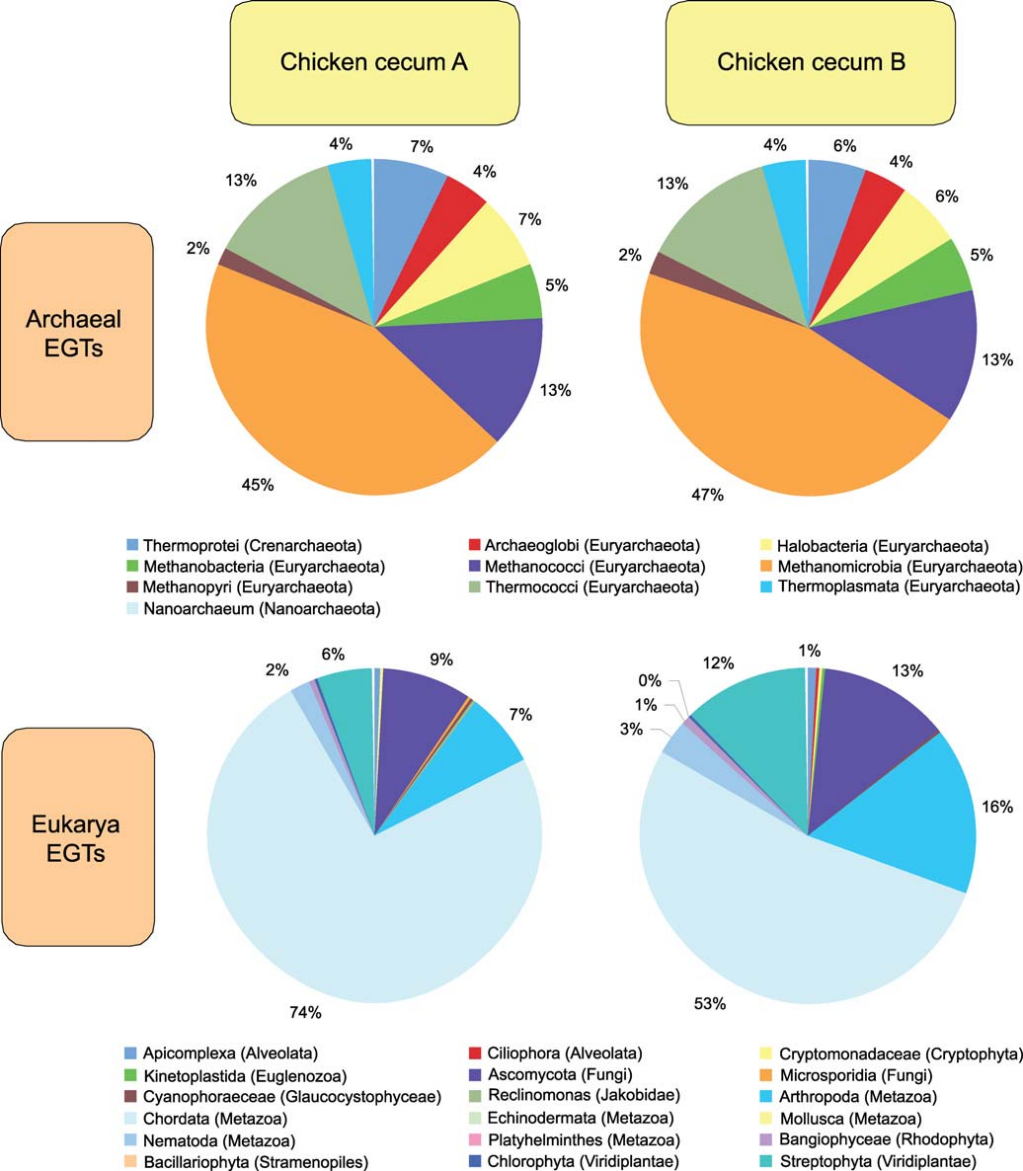
Phylogenetic composition of archaeal and eukaryotic environmental gene tags (EGTs) from two pyrosequenced chicken cecum samples. The percent of EGTs in each of the archaeal class or eukaryotic division from the two pyrosequenced chicken cecum samples microbiomes is shown.

Bacterial specific EGTs represented approximately 97% of the total EGTs (Table 1) and the distribution of phylotypes fell predominantly into the *Firmicutes*, *Bacteroidetes* and *Proteobacteria* groups, regardless of the microbiome analyzed (Figure 1). The distribution of EGTs from the Bacteria is congruent with the distribution of SSU rDNA phylotypes, as was found with the Soudan Mine and rumen microbiome studies [10], [39]. Archaeal EGTs constituted approximately 1% of EGTs in these metagenome libraries (Table 1), matching well with previous estimates of Archaea numbers in the adult chicken cecum microbiome [23], [24]. The majority of Archaeal EGTs correspond to methanogenic classes with the largest proportion corresponding to the Euryarchaeota (Figure 3). The majority of eukaryotic EGTs (75 and 53%, respectively) were most similar to the *Chordata* (i.e., host), fungi (6 and 12%, respectively) and the *Viridiplantae* (i.e., feed; 6 and 12%, respectively) (Figure 3). These EGT proportions were expected from our current knowledge of the chicken cecum microbiome community structure.

We also used two independent statistical analyses to measure the diversity in these microbiomes (Table 2). First, we applied Shannon-Weiner, Simpson's lambda, and Pielou's evenness analyses for measuring species richness and evenness [43] for the SSU rDNA hits against the European Ribosome Database. We also used the PHACCS analysis system [44] to estimate the genotype richness, diversity, and evenness of the different metagenomes by analyzing random sequences in the two microbiomes (Table 2). The cecum A microbiome had less richness and evenness than the cecum B microbiome regardless of the statistical model. The community structure changes from logarithmic (chicken cecum A) to lognormal (chicken cecum B). In chicken cecum A compared with chicken cecum B, there are a great number of species (richness; ∼3,500 genotypes compared to ∼1,900 genotypes), but a higher dominance of some genotypes.

**Table 2:** Diversity analysis of the chicken cecum microbiomes.

SAMPLE	SSU rDNA Richness	SSU rDNA Shannon Wiener Index	SSU rDNA Pielou Evenness	SSU rDNA Simpson Evenness Index	Random Sequence Richness	Random Sequence Evenness	Random Sequence Most Abundant Genotype (%)	Random Sequence Shannon Wiener Index
Cecum A	160 genotypes	4.074	0.803	0.944	1,908 genotypes	0.9804	1.84	7.41 nats
Cecum B	179 genotypes	4.795	0.924	0.987	3,522 genotypes	0.9614	0.39	7.85 nats

**Figure 4:**
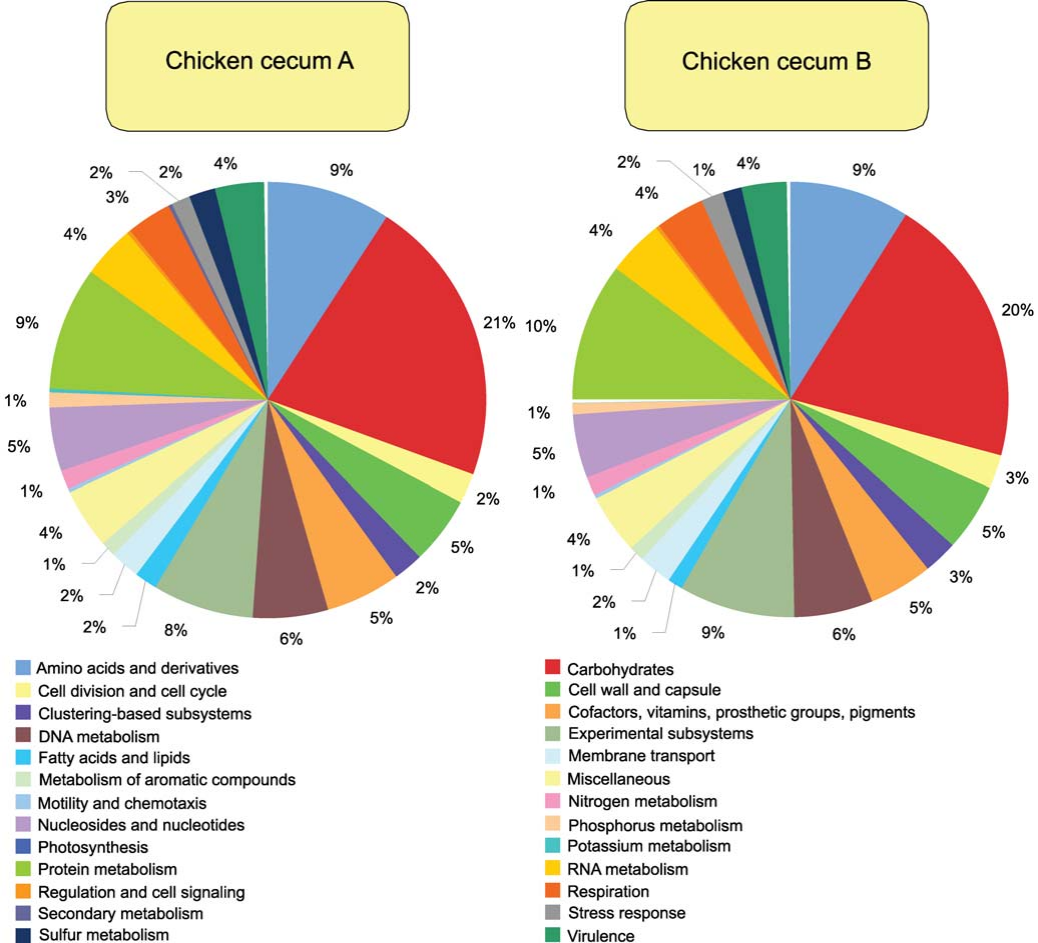
SEED subsystem composition of chicken cecum A and B microbiomes is shown. The percent of environmental gene tags (EGTs) in each of the SEED subsystems from the chicken cecum A and B microbiomes is shown. The BLASTX cutoff for EGTs is 1×10^−5^.

**Figure 5 pone-0002945-g005:**
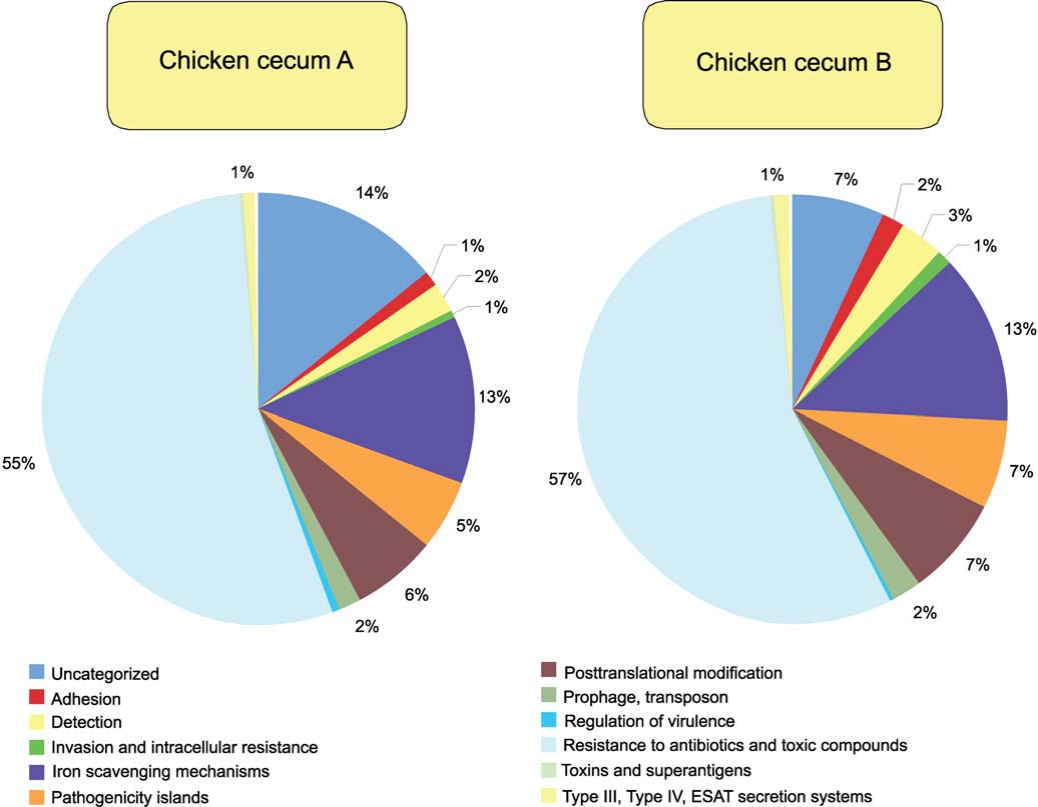
Virulence subsystem composition of chicken cecum A and B microbiomes is shown. The percent of environmental gene tags (EGTs) in each of the virulence subsystems from the chicken cecum A and B microbiomes is shown. The BLASTX cutoff for EGTs is 1×10^−5^.

The subsystems-based annotations (SEED) database was utilized to gain a better understanding of these phylogenetic trends and to predict the metabolic potential (content of EGTs) of these microbiomes (Figures 4–8). The EGT proportions were also expected from our current knowledge of the cecal microbiome community structure. The subsystems are annotated across genomes and are based on biochemical pathways, fragments of pathways, and clusters of genes that function together, or any group of genes considered to be related. Much of this analysis is dependent on sequence databases, and while we tried to avoid database bias by using multiple databases and alternative querying algorithms for analysis, we are aware that some sequences have no matched relatives in the databases, or are over-represented in the databases. Further, sequence similarity does not always mean functional similarity and this may influence the interpretation of our results as minor sequence dissimilarities may represent functional different or even a completely new functions. Consistent with our analysis of 45 microbiomes [12], the chicken cecum microbiomes are dominated by carbohydrate metabolism, and are sparsely populated with genes for respiration, reflecting the more stable anoxic environment in the gastrointestinal tract. Genes associated with the cell wall metabolism were abundant, as were virulence genes (Figure 5). To extend this analysis, we applied statistical methods [45], which compare those subsystems that are more, or less, represented in the different microbiomes (sample size of 5,000 proteins, 20,000 repeated samples; p<0.02). Again, consistent with the higher abundance of *Bacteriodetes* within cecum A, this metagenome had higher levels of the following subsystems when compared with cecum B; Chitin and N-Acetylglucosamine Utilization, L-Arabinose Utilization, L-Rhamnose Utilization, Lactose Utilization, Conjugative Transposon from Bacteroidales, Galactosylceramide and Sulfatide Metabolism, and Ton and Tol Transport Systems.

**Figure 6 pone-0002945-g006:**
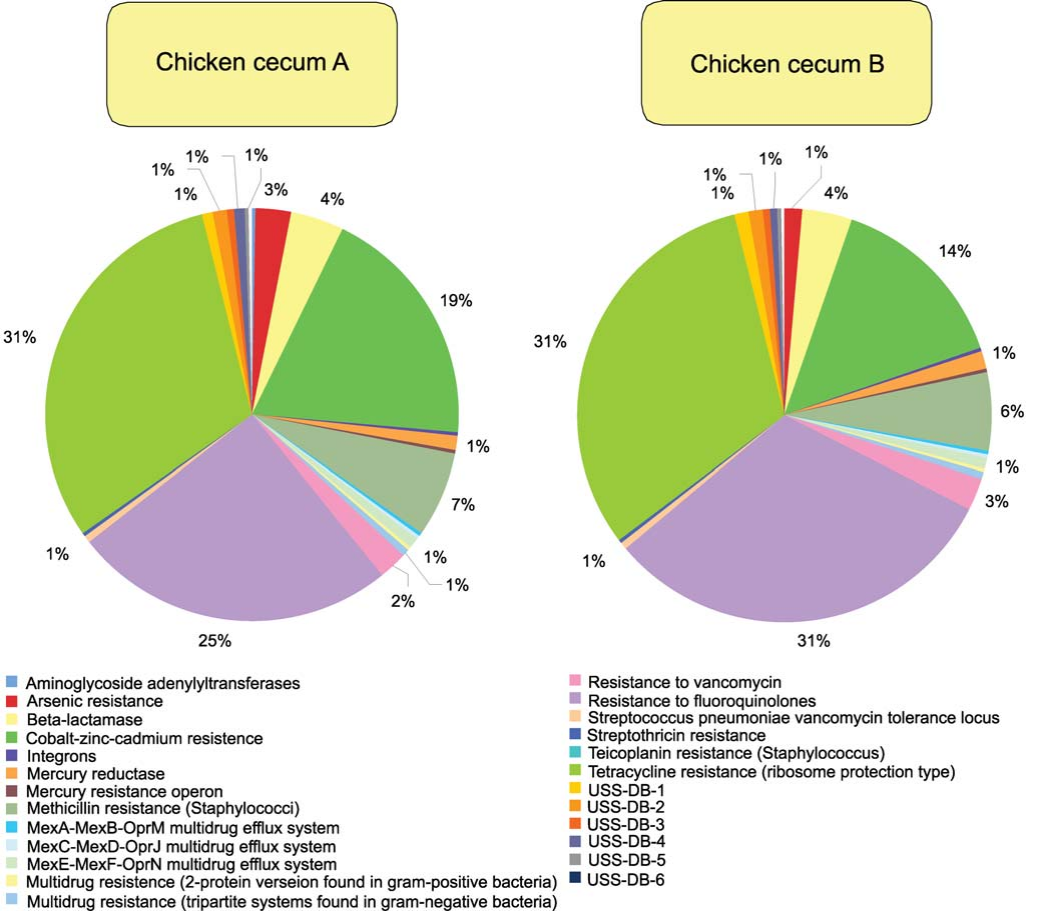
Resistance to antibiotics and toxic compounds subsystem composition of chicken cecum A and B microbiomes is shown. The percent of environmental gene tags (EGTs) in each of the Resistance to antibiotics and toxic compounds subsystems from the chicken cecum A and B microbiomes is shown. The BLASTX cutoff for EGTs is 1×10^−5^.

**Figure 7 pone-0002945-g007:**
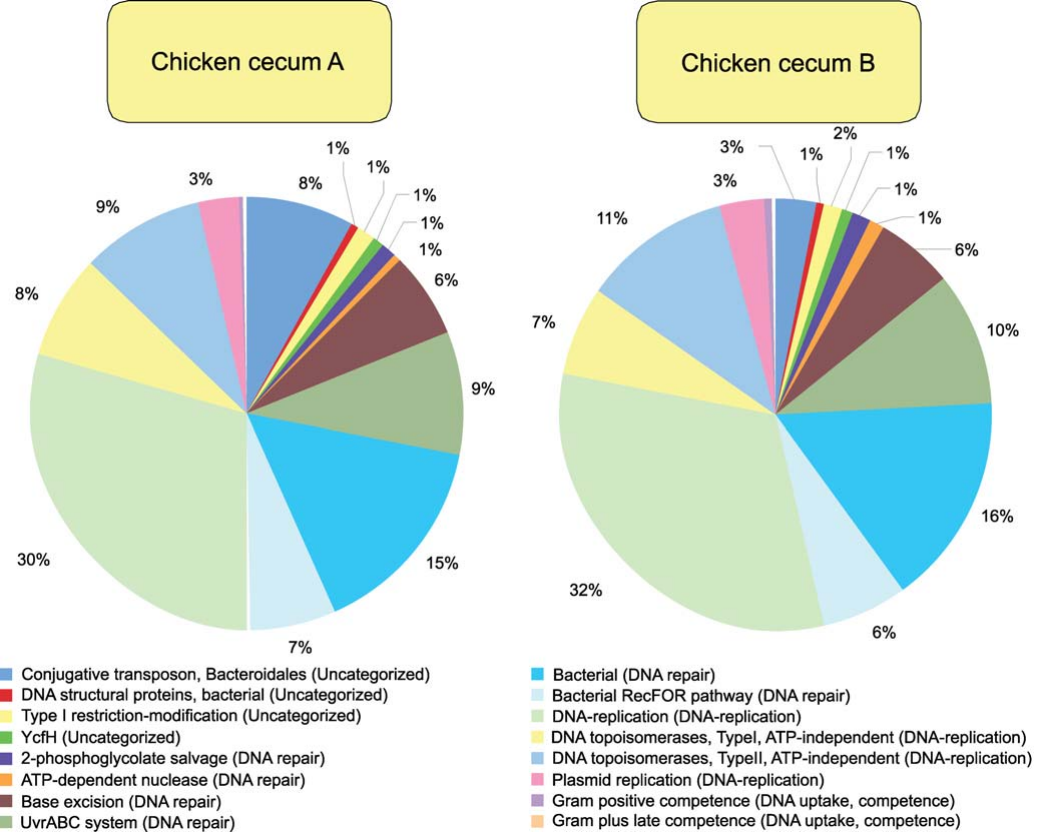
DNA Metabolism subsystem composition of chicken cecum A and B microbiomes is shown. The percent of environmental gene tags (EGTs) in each of the DNA Metabolism subsystems from the chicken cecum A and B microbiomes is shown. The BLASTX cutoff for EGTs is 1×10^−5^.

**Figure 8:**
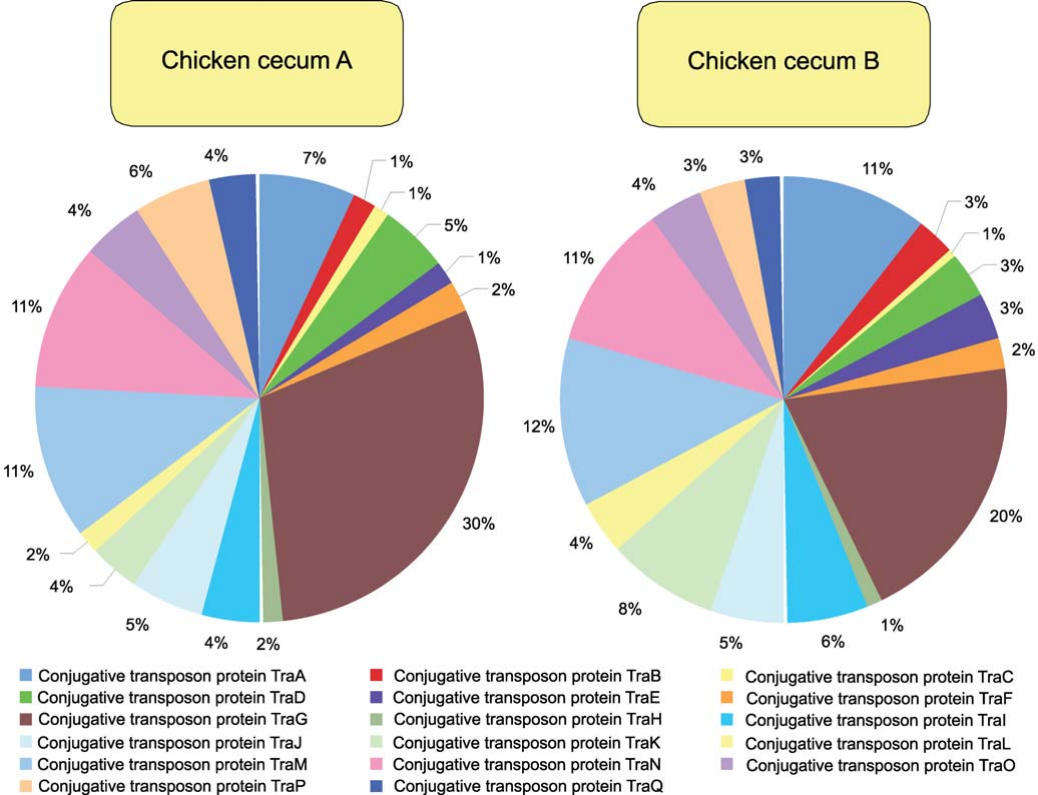
Conjugative transposon, *Bacteriodales* subsystem composition of chicken cecum A and B microbiomes is shown. The percent of environmental gene tags (EGTs) in each of the Conjugative transposon, Bacteriodales subsystems from the chicken cecum A and B microbiomes is shown. The BLASTX cutoff for EGTs is 1×10^−5^.

When looking solely at the chicken cecum and the SEED Virulence Subsystem, resistance to antibiotics and other toxic compounds dominated (55–57%). Resistance to both tetracyclines and fluoroquinolones represented 25 to 31% of the EGTs in this subsystem (Figure 6). Cobalt-zinc-cadmium resistance was also found to be abundant. These antibiotics are used routinely in poultry production and so their presence is not unexpected, even though their abundance is striking with respect to the other classes of virulence genes. The other class of genes, found in both the DNA metabolism and the virulence categories, are those genes associated with *Bacteroidales* conjugative transposons or mobile DNA elements which are detected in similar numbers to those of tetracycline resistance (Figures 7 and 8). Consistent with the higher abundance of *Bacteriodetes* within Cecum A, this metagenome had higher levels of the *Bacteroidales* conjugative transposon (Wilcoxon exact test P = 0.021) compared with cecum B, and the difference was driven by a higher proportion of TraG within this metagenome (Wilcoxon exact test, P<0.001).

**Figure 9:**
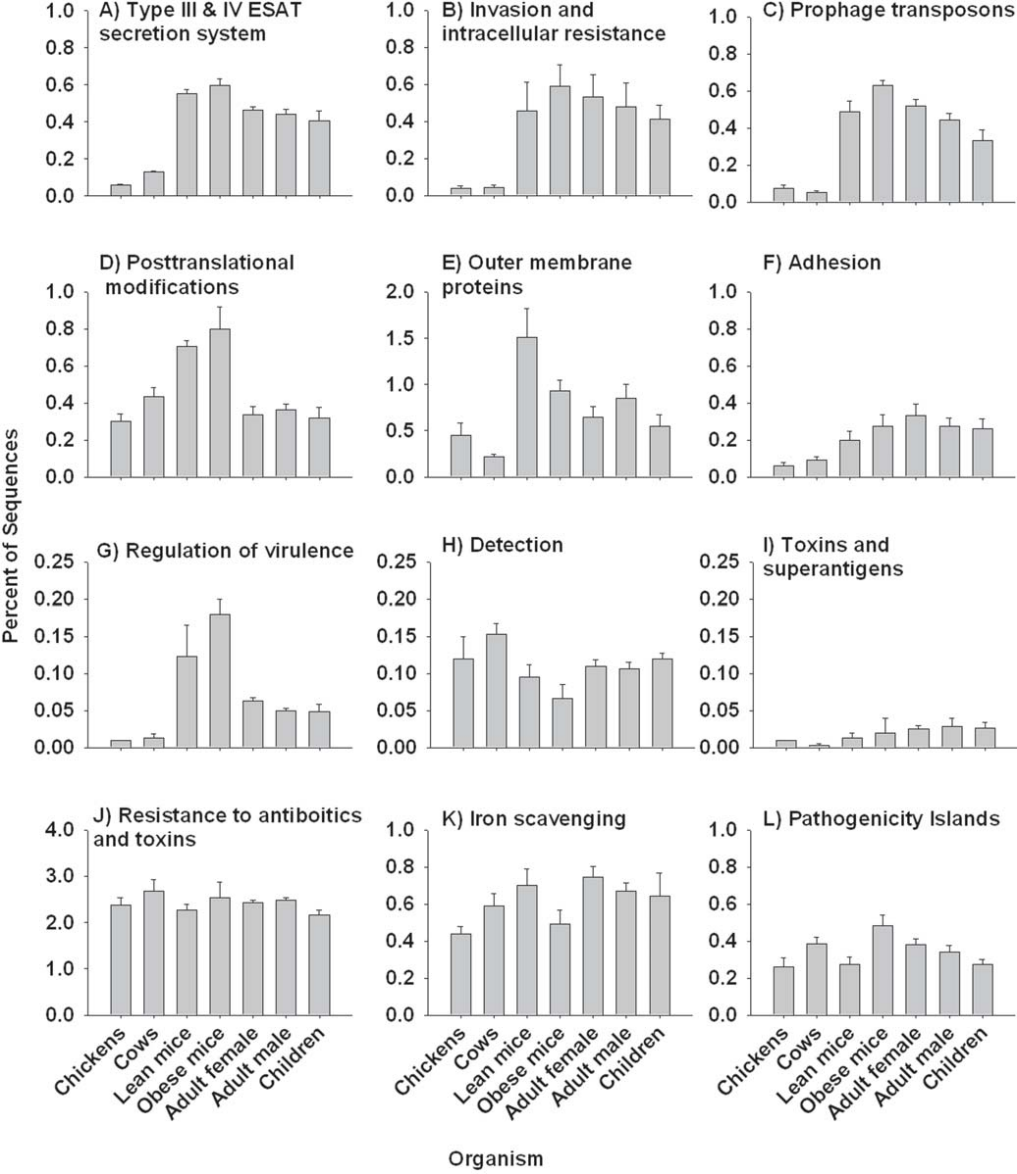
The mean (SE) percent of sequences identified within the SEED Virulence Subsystem in the microbiomes from chicken cecum, bovine rumen, mouce cecum and human fecal samples.

We then compared the twelve major pathways in the SEED Virulence Subsystem represented in the chicken cecum (two samples by 454 pyrosequencing), bovine rumen (four samples by 454 pyrosequencing) [39], mouse cecum (5 samples by Sanger sequencing and two samples by 454 pyrosequencing) [11] and human fecal microbiomes (15 samples by Sanger sequencing) [46], [47] by a multivariate analysis of variance (MANOVA) using on the percent of sequences showing similarity to each pathway (Figure 9). The chicken cecum and bovine rumen metagenomes had lower abundances of Adhesion (F_6_ = 3.135, P<0.001), Prophage transposons (F_6_ = 17.335, P<0.001), and Invasion and Intracellular Resistance (F_6_ = 5.297, P = 0.001) EGTs. In contrast, EGTs in the Regulation of Virulence subsytem (F_6_ = 8.691, P<0.001) and Type III and IV ESAT secretion systems (F_6_ = 21.886, P<0.001) were low in chicken cecum and bovine rumen, but higher in the human fecal microbiomes, and with even a higher representation in the mouse cecal microbiomes. Mouse cecal microbiome contained more outer membrane proteins (F_6_ = 6.189, P<0.001), and Posttranslational Modification (F_6_ = 11.302, P<0.001) EGTs than the other micrbiomes and the Detection subsystem was higher in bovine rumen when compared with the other microbiomes (F_6_ = 3.888, P = 0.009). Pathogenicity islands were higher in the obese mice cecal microbiomes when compared to other microbiomes (F_6_ = 3.851, P = 0.009). There were no differences in EGT content within these microbiomes in the following subsystems; Iron scavenging mechanism (F_6_ = 1.03, P = 0.43), Resistance to antibiotics and toxic compounds (F_6_ = 1.406, P = 0.258), Toxins and superantigens (F_6_ = 1.042, P = 0.427).

**Figure 10:**
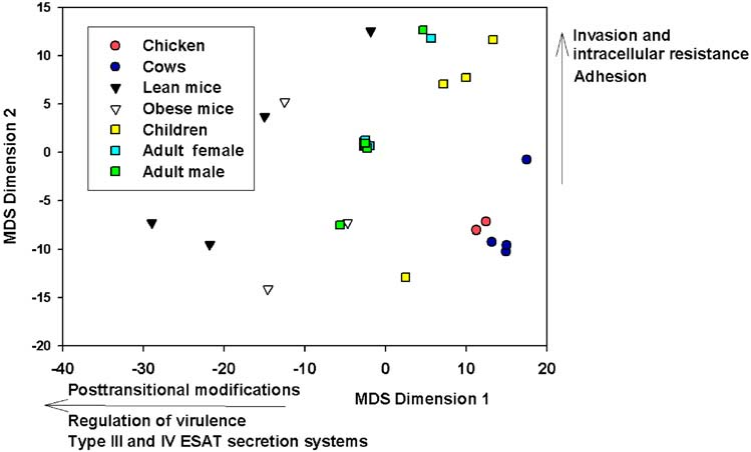
A multi-dimensional representation of the SEED Virulence Subsystem EGTs in the microbiomes from chicken cecum, bovine rumen, mouce cecum and human fecal samples. The groups were divided to create similar group sizes which ensures better statistical outcomes. Each subsystem was tested for normality and log transformed where required. A General Linear Model was used with a post hoc Tukey's test being used to identify group membership. The differences between the subsystem abundance in each organism were then visualized using proxscal multidimensional scaling (MDS). The MDS was conducted on a single start and required 594 iterations, with Stress value of 0.102. The arrows indicate the direction in which the proportion of sequences was increasing and was driving the separation between metagenomes.

After a hierarchal clustering analysis, non-dimensional scaling was then used to determine the relationship between these the metavirulome of these microbiomes (Figure 10). The abundance of four virulence pathways differed between organisms and are the driving factors in the metavirulome clustering. Microbiomes from chicken cecum and bovine rumen showed a low abundance of EGTs showing similarity to the Type III and IV ESAT Secretion System, Invasion and Intracellular Resistance, Prophage Transposons, and Adhesion and Regulation of Virulence subsystems. The mouse cecal microbiomes showed the widest level of variation in the abundance of sequences similar to each subsystem, regardless of sequencing technology. The adult male and female humans had remarkable similarity in the abundance of sequences to each subsystem, except for Male InA which was more similar to the mouse cecal microbiome due to higher abundances of sequences similar to outer membrane proteins. The two human subjects from the USA [46] were most similar to each other, and were not similar to the other adult human samples from Japan [47]. The human fecal microbiomes from the two weaned children were similar to the adult signature. The sample from Child F1U was an extreme outlier and this possibly caused by low levels of EGTs that showed similarity to the Adhesion and Posttransitonal Modification subsystems.

**Figure 11:**
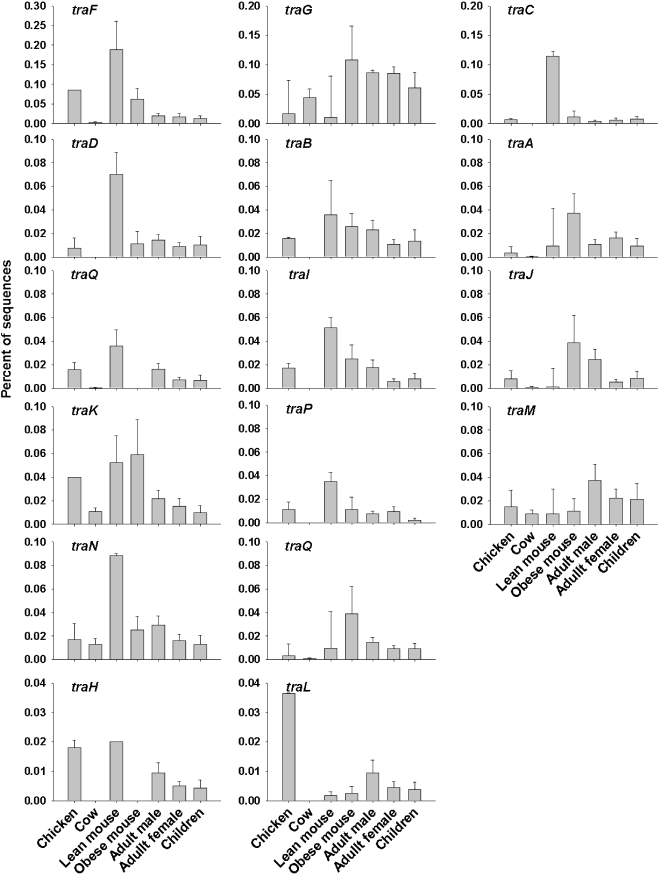
The distribution of sequences similar to each transposon gene from the chicken cecum, mouse cecum, human fecal and rumen microbiomes.

The number of sequences that showed similarity to the *Bacteroides* transposon group was 541 and 159 in Chicken cecum A and B respectively, suggesting that they are worthy of investigation. Chicken cecum metagenomes had the broadest range of genes within the conjugative transposon subsystem, with 17 genes represented, however the mice cecum microbiomes had a higher abundance of sequences similar to transposons. In comparison the human fecal metagenomes only carried one transposon gene, *traF*. The lean mouse cecal microbiome had an average of 10.6 genes and obese mouse cecal microbiome had an average of 12 genes represented. One mouse, lean mouse 1, had the highest abundance of transposon genes. The number of genes represented across the whole dataset was low, making normalization of the data difficult. Thus, a non-parametric pairwise T-test was used to describe the difference between the individual microbiomes. Two genes *traE* and *traA* were only present in the chicken cecum metagenomes. The distribution of transposon genes between the two chicken cecum microbiomes and those in the mouse cecum, human fecal and rumen microbiomes also differed (Table 3 and Figure 11). Chicken cecum A was particularly over-represented was *traF*, *traO* and *traQ* in comparison with Chicken cecum B. In general, the chicken cecum microbiomes contained a different complement of transposon genes from the rumen and obese mouse cecum microbiomes. Chicken cecum B was different to all mouse cecal metagenomes, due to the low abundance of transposon genes. The lean mouse 1 cecal microbiome was overrepresented with *traF*, *traP*, *traM*, *traG*, *traL*, *traH* and was different compared to all other metagenomes. The other mice cecal microbiomes had a similar distribution of transponson sequences. Interestingly, the human fecal microbiomes had either few transposon genes or many transposon genes from this gene family. Because of this, the human fecal microbiomes, with few transposon genes, differed from the chicken cecum microbiomes, whereas the human fecal microbiomes, possessing many transposon genes, were similar to the chicken cecum microbiomes.

**Table 3:** The results of a Wiloxon test to compare the abundance of Transposon genes in the chicken cecum, mouse cecum, human fecal and rumen microbiomes.

Chicken A	Z Score	Significance	Chicken B	Z score	Significance
Chicken A vs. chicken B	−3.527	0.000			
Human 7 (13665)	−3.623	0.000	HumaninM	−3.625	0.000
Human8	−3.623	0.000	Rumen 710F6 160698	−3.623	0.000
HumaninE	−3.623	0.000	Human F2 Y 44637	−3.622	0.000
HumaninM	−3.623	0.000	Human F1 S 36048	−3.575	0.000
Rumen 710F6 (160698)	−3.622	0.000	Human F2 W 38078	−3.480	0.001
Rumen 80F6 (47885)	−3.622	0.000	Mouse obese 1 3143	−3.290	0.001
Rumen pooled planktonic (60955)	−3.622	0.000	Human In A 25759	−3.290	0.001
Rumen 640F (662531)	−3.575	0.000	Mouse lean 1 3048	−3.148	0.002
Mouse obese 454	−3.480	0.001	HumaninE	−3.009	0.003
Human F1 T 42144	−3.480	0.001	Rumen 640F6 62531	−2.912	0.004
Human F2 V 44394	−3.385	0.001	Human 7 13665	−2.819	0.005
Human In R 40935	−3.007	0.003	human8	−2.819	0.005
Mouse lean 1 (3048)	−2.675	0.007	Rumen pooled planktonic 60955	−2.818	0.005
Mouse lean 454	−2.580	0.010	Rumen 80F6 47885	−2.676	0.007
Human F2 Y 44637	−2.107	0.035	Human F2 X 37249	−2.580	0.010
Mouse obese 1 (3143)	−1.918	0.055	Human In D 44036	−2.438	0.015
Human F2 X 37249	−1.681	0.093	Mouse obese 454	−2.249	0.025
Human F1 S 36048	−1.160	0.246	Mouse lean 2 2828	−2.155	0.031
Mouse lean 3 (2894)	−1.018	0.309	Mouse lean 3 2894	−1.870	0.061
Human In A 25759	−0.686	0.492	Human F1 T 42144	−0.545	0.586
Human F2 W 38078	−0.450	0.653	Mouse lean 2 2828	−0.544	0.586
Mouse obese 2 (2438)	−0.118	0.906	Mouse lean 454	−0.260	0.795

While a limitation of the random sample pyrosequencing approach is the resulting short read lengths, we were able to assemble some of these reads into 33 contigs of >500 nucleotides (32 from cecum A and one from cecum B; Table 4 and Table 5). Translations of these contigs (EGTs) were used for BLASTX analysis. The majority of these translations showed similarity with genes from the *Bacteroidetes* (20 contigs), the dominant taxa from this microbiome. Seven contigs shared amino acid sequence similarities (54 to 100%) with transposases from the *Bacteroidetes*, confirming the results from the non-assembled data, two contigs shared sequence similarity (99 and 100%, respectively) with proteases from the *Bacteroidetes*, and seven contigs had sequence similarity with hypothetical proteins found in *Bacteroidetes*. In addition, there were single contig matches for xyulose kinase and L-rhamnose/H+ symporter also from the *Bacteroidetes*. Finally, there was one contig that exhibited 92% sequence similarity with the BcrA drug efflux gene from *Enterococcus faecalis*. The single assembled contig from chicken cecum B showed 93% amino acid sequence similarity with a hypothetical protein from *Bacteroides ovatus*.

**Table 4:** Summary of blastx results of chicken cecum A assembled contigs.

Contig	Length	Genbank #	Annotation	Organism	Score	E value (Expect)	Identities (%)
00246	1552	YP_001298993.1	putative transposase	*Bacteroides vulgatus*	483	1e-134	99
		ZP_01958971.1	hypothetical protein BACCAC_00562	*Bacteroides caccae*	410	2e-112	96
		ZP_01958551.1	hypothetical protein BACCAC_00123	*Bacteroides caccae*	407	8e-112	95
12082	629	YP_001304583.1	putative transposase	*Parabacteroides distasonis*	328	1-88	98
		YP_001297372.1	putative transposase	*Bacteroides vulgatus*	274	3e-72	100
		ZP_01961195.1	hypothetical protein BACCAC_02821	*Bacteroides caccae*	180	6e-44	74
25141	526	YP_001300792.1	putative transposase	*Bacteroides vulgatus*	231	2e-59	100
		YP_001297547.1	putative transposase	*Bacteroides vulgatus*	229	4e-59	99
		YP_001298735.1	putative transposase	*Bacteroides vulgatus*	229	4e-59	99
25921	921	YP_001297694.1	transposase	*Bacteroides vulgatus*	572	2e-161	100
		YP_001298173.1	transposase	*Bacteroides vulgatus*	570	4e-161	99
		ZP_01890000.1	transposase	*unidentified eubacterium*	280	9e-74	46
25932	1045	YP_001297547.1	putative transposase	*Bacteroides vulgatus*	640	0.0	99
		YP_001300792.1	putative transposase	*Bacteroides vulgatus*	640	0.0	100
		NP_812469.1	putative transposase	*Bacteroides thetaiotaomicron*	640	0.0	99
25980	1447	ZP_01254245.1	transposase	*Psychroflexus torquis*	352	4e-95	54
		ZP_01890000.1	transposase	*unidentified eubacterium*	345	6e-93	53
		YP_001298173.1	transposase	*Bacteroides vulgatus*	306	2e-81	46
26741	583	YP_001297710.1	transposase	*Bacteroides vulgatus*	396	4e-109	99
		YP_001297386.1	transposase	*Bacteroides vulgatus*	396	4e-109	99
		EDO11111.1	hypothetical protein BACOVA_03013	*Bacteroides ovatus*	388	9e-107	96
09258	606	YP_001299838.1	conserved hypothetical protein, putative TonB	*Bacteroides vulgatus*	253	2e-82	99
		YP_213356.1	hypothetical protein BF3767	*Bacteroides fragilis*	67.8	4e-10	37
		YP_101268.1	TonB	*Bacteroides fragilis*	67.8	4e-10	37
10937	607	YP_001297387.1	hypothetical protein BVU_0035	*Bacteroides vulgatus*	145	7e-52	90
		EDO11113.1	hypothetical protein BACOVA_03015	*Bacteroides ovatus*	140	1e-33	94
		EDO11112.1	hypothetical protein BACOVA_03014	*Bacteroides ovatus*	84	6e-15	84
25918	874	EDO52987.1	hypothetical protein BACUNI_02999	*Bacteroides uniformis*	160	9e-38	97
		EDO13931.1	hypothetical protein BACOVA_00321	*Bacteroides ovatus*	155	4e-36	95
		EDO11072.1	hypothetical protein BACOVA_02972	*Bacteroides ovatus*	155	4e-36	95
25929	639	EDO53852.1	hypothetical protein BACUNI_02471	*Bacteroides uniformis*	129	2e-28	93
		ZP_01962139.1	hypothetical protein BACCAC_03787	*Bacteroides caccae*	127	4e-28	92
		YP_001152206.1	ORF137	*Pinus koraiensis*	93.6	8e-18	56
25961	1554	CAJ30045.1	conserved hypothetical protein	*Magnetospirillum gryphiswaldense*	141	1e-31	54
		EDO11070.1	hypothetical protein BACOVA_02970	*Bacteroides ovatus*	107	2e-21	65
		EDO53850.1	hypothetical protein BACUNI_02467	*Bacteroides uniformis*	107	3e-21	83
26526	1002	EDP10382.1	hypothetical protein EUBDOL_01624	*Eubacterium dolichum*	228	6e-58	100
		EDP10383.1	hypothetical protein EUBDOL_01625	*Eubacterium dolichum*	219	3e-55	100
		EDP10384.1	hypothetical protein EUBDOL_01626	*Eubacterium dolichum*	205	3e-51	100
27457	1346	EDO51852.1	hypothetical protein BACUNI_04402	*Bacteroides uniformis*	321	8e-86	73
		EDO55703.1	hypothetical protein BACUNI_00736	*Bacteroides uniformis*	232	4e-59	77
		EDO51853.1	hypothetical protein BACUNI_04403	*Bacteroides uniformis*	143	1e-54	56
22050	614	YP_001298764.1	transcriptional regulator	*Bacteroides vulgatus*	47	8e-04	100
		ZP_01960532.1	hypothetical protein BACCAC_02149	*Bacteroides caccae*	43.9	0.006	72
		YP_133705.1	MobN1	*Bacteroides uniformis*	40.4	0.071	68
25094	552	YP_001298467.1	putative exported protease	*Bacteroides vulgatus*	374	2e-102	95
		ZP_02033115.1	hypothetical protein PARMER_03138	*Parabacteroides merdae*	244	2e-63	62
		YP_212141.1	putative exported tricorn protease	*Bacteroides fragilis*	239	5e-62	60
00991	554	YP_001297716.1	hypothetical protein BVU_0375	*Bacteroides vulgatus*	338	7e-92	99
		YP_001304010.1	hypothetical protein BDI_2669	*Parabacteroides distasonis*	210	3e-53	62
		YP_213505.1	hypothetical protein BF3924	*Bacteroides fragilis*	199	9e-50	55
26290	647	XP_454253.1	unnamed protein product	*Kluyveromyces lactis*	35	3.4	30
		XP_453624.1	unnamed protein product	*Kluyveromyces lactis*	34.7	4.4	31
		NP_197442.1	ABC transporter family protein	*Arabidopsis thaliana*	33.5	9.9	32
25938	583	AAS78451.1	BcrA	*Enterococcus faecalis*	355	7e-97	92
		YP_001273166.1	multidrug ABC transporter, ATPase component, CcmA	*Methanobrevibacter smithii*	224	2e-57	58
		YP_001086833.1	putative lantibiotic ABC transporter,ATP-binding protein	*Clostridium difficile*	221	3e-56	56
25946	541	YP_001299600.1	xylulose kinase	*Bacteroides vulgatus*	370	2e-101	100
		EDO54840.1	hypothetical protein BACUNI_01362	*Bacteroides uniformis*	345	8e-94	91
		EDO12568.1	hypothetical protein BACOVA_01710	*Bacteroides ovatus*	337	2e-91	88
26189	591	YP_001297927.1	L-rhamnose/H+ symporter	*Bacteroides vulgatus*	392	5e-108	100
		NP_812676.1	L-rhamnose/H+ symporter	*Bacteroides thetaiotaomicron*	374	2e-102	93
		EDO10968.1	hypothetical protein BACOVA_03603	*Bacteroides ovatus*	372	6e-102	93
27252	545	YP_001301101.1	dipeptidyl aminopeptidase	*Bacteroides vulgatus*	375	7e-103	100
		ZP_02031298.1	hypothetical protein PARMER_01283	*Parabacteroides merdae*	322	5e-87	81
		YP_001303599.1	dipeptidyl aminopeptidase	*Parabacteroides distasonis*	308	1e-82	77
03484	541	YP_001298244.1	putative transmembrane protein	*Bacteroides vulgatus*	201	1e-50	100
		YP_211132.1	putative transmembrane protein	*Bacteroides fragilis*	87	5e-16	59
		YP_211133.1	putative transmembrane protein	*Bacteroides fragilis*	81.3	3e-14	77

**Table 5:** BlastX alignments for the chicken cecum A assembled contigs.

Contig 00246	MIAIIKGTDVHTVTSVLLKLSRRRRYQVREITLDMAPNMEQIARICFPAAKRVTDRFHVQKLAYEAVQEMRVKARWEALDEESTQIAYAKACGKMYHAPVFANGDTR
YP_001298993	MIAIIKGTDVHTVTSVLLKLSRRRRYQVREITLDMAPNMEQIARICFPAAKRVTDRFHVQKLAYEAVQEMRVKARWEALDEESTQIAYAKACGKMYHAPVFANGDTR
Contig 00246	KQLLARSIYLLYKKESLWTQSQRIRAEILFKEYPDIKKGYYLSMRLGLIYHQCKFKDIALTRLARWYDEVDKSGFLTFGRVARSIQTHYLDIINFFERRATNAAAE
YP_001298993	KQLLARSIYLLYKKESLWTQSQRIRAEILFKEYPDIKKGYYLSMRLGLIYHQCKFKDIALTRLARWYDEVDKSGFLTFGRVARSIQTHYLDIINFFERRATNAEAE
Contig 00246	SFNAKIKAFRAQFRGVRDRAFFLYRLAKLYA
YP_001298993	SFNAKIKAFRAQFRGVRDRAFFLYRLAKLYA
Contig 12082	MFPESKVTEIYCMADDFCKEFTLQQEKYMIKDMKTMHRNKPNRMSDAEIMVILILFHSGGFRCFKHYYKEYVCKHLKHLFPRQVSYNRFVELEKEVLLPMTIFIK
YP_001304583	MFPESKVTEIYCMADDFCKEFTLQQEKYMIKDMKTMHRNNPNRMSDAEIMVILILFHSGGFRCFKHYYKEYVCKHLKHLFPRQVSYNRFVELEKEVLLPMTIFIK
Contig 12082	RVLLGTCTGISFVDSTPLCVCRNQRILIHKTFEGLAERGRCSMGWFFGFKLH
YP_001304583	RVLLGTCTGISFVASTPLCVCRNQRILIHKTFEGLAERGRCSMGWFFGFKLH
Contig 25141	MQQKLMNVRVRCVAADSIYANNANRKFCTKYGISTSFVRKGRAAKDEPLRKVLRSELSKERATRLEGSFGTQKQHYSLSRIKARNRKTEILWIFFGIHTANAIL
YP_001300792	MQQKLMNVRVRCVAADSIYANNANRKFCTKYGISTSFVRKGRAAKDEPLRKVLRSELSKERATRLEGSFGTQKQHYSLSRIKARNRKTEILWIFFGIHTANAIL
Contig 25141	IIEKIRNKTAKAA
YP_001300792	IIEKIRNKTAKAA
Contig 25921	LEHAHDYLLYPENIGENLSLDETCLSNGDVYTILTNKAAKGRKGALVAMVRGVATDAVSGILRRLPHRKRLSVKTVTTDLSSAMMLTVRKVFPAAKLINDRFHV
YP_001297694	LEHAHDYLLYPENIGENLSLDETCLSNGDVYTILTNKAAKGRKGALVAMVRGVATDAVSGILRRLPHRKRLSVKTVTTDLSSAMMLTVRKVFPAAKLINDRFHV
Contig 25921	QQLMSEAVDRLRIRYRWKVLDAENQAIREHRQkkkeakskaereRIGKWEPERMENGETLPQIVSRSKHIILKHWSKWNEQQKTRAAILFDKFPKLLEGYSLSM
YP_001297694	QQLMSEAVDRLRIRYRWKVLDAENQAIREHRQKKKEAKSKAERERIGKWEPERMENGETLPQIVSRSKHIILKHWSKWNEQQKTRAAILFDKFPKLLEGYSLSM
Contig 25921	KLTDIFNKKSGPDEARLNLARWYNEVEKFDYMEFNKVLDTFSNHSTTIINYFEERLTNASAESFNAKIKAFRSQLRGVADLKFFMFRLARLYA
YP_001297694	KLTDIFNKKSGPDEARLNLARWYNEVEKFDYMEFNKVLDTFSNHSTTIINYFEERLTNASAESFNAKIKAFRSQLRGVADLKFFMFRLARLYA
Contig 25932	MAKIVNISEIHPTLGFTEFDILEKYRKSFNESELGKLHSVFPFECMAKAAGLSDRRLGRRNRFSPSAKIALMVLKAYTGFSDRQLVEHLNGNIHYQIFCGIMI
YP_0012297547	MAKIVNISEIHPTLGFTEFDILEKYRKSFNESELGKLHSVFPFECMAKAAGLSDRRLGRRNRFSPSAKIALMVLKAYTGFSDRQLVEHLNGNIHYQIFCGIMI
Contig 25932	PPSLPITNFKIVSAIRNEIASRLDIDSFQELLASHWKPYLDNLHVCMTDATCYESHMRFPTDMKLLWESLEWLYRHICRHCRELGIRRPRNKYRNVAESYLSY
YP_0012297547	PPSLPITNFKIVSAIRNEIASRLDIDSFQELLASHWKPYLDNLHVCMTDATCYESHMRFPTDMKLLWESLEWLYRHICRHCRELGIRRPRNKYRNVAESYLSY
Contig 25932	CkkrkrrasrarmlkrrMIKLLEKLLSQRDGIHSEYGALLRYTQDYHKRLSIIRKVLVQEKEMFEGRKVSDRIVSIDRHYVRPIVRGKETKSVEFGAKVNNIQ
YP_0012297547	CKKRKRRASRTRMLKRRMIKLLEKLLSQRDGIHSEYGALLRYTQDYHKRLSIIRKVLVQEKEMFEGRKVSDRIVSIDRHYVRPIVRGKETKSVEFGAKVNNIQ
Contig 25932	IDGISFIEHLSFKAFNEGIRLKDCI
YP_0012297547	IDGISFIEHLSFKAFNEGIRLKDCI
Contig 25980	YYHINGDTFEKQYKEVLSGYREWSELSHAEDWLVFPENIGESICIDETAPSNGELYTIVSNRSSRGGKGTIIAIVKGVAADAVTEALMRI DEDKRLLVKEITMDM
ZP-01254245	YYKINGRILQYHYKNHLSDFKDWIQKEHAQDWLLYPENIGTYLSLDETSLSNGELYTILTNKNAQGKKGSIVAIVKGTRAIDVINILNKI PLERRNVVEEVTVDM
Contig 25980	SNSMRLIARRCFPNAMRTIDRFHIQKLACDALQEMRIAHRWDAIQADTDAREEAKCLGEAYTPIVLANGDTHKQLLARSRYLLFKSADKWTESQRQRAEVLFETY
ZP-01254245	AGSMNLIAKKCFPKTELVTDRFHVQKLASEAVQEERIRLRWEIMEQENSDILEARKKGRTYKFELLGNGDTHKQLLARSRYLLFKSKTKWTVRQRERAEILFKLY
Contig 25980	PDLKEAYSLTHSLRMIFSKNTVKDAARLSLARWYNKVDDSGFKSFNVIAATLYEHYDEVLNFFVNRATNAFAESFNAKIKAFRAALRGVTDIKFFLFRLTKLYA
ZP-01254245	PSIEKAYNLAQGLTYIFENNTHKDVARLKLAHWYDKVEKSQFKSFSTIARSIQMHYVPILNYFNNRSTNASAESFNAKIKEFRAQFRGVRDVKFFLYRLTKLFA
Contig 26741	PMPEGLSLEGATKLGEEVSEQYAVSPARFYVKRIIRPKYRLADGRIITAPMPVMAHPHSNASESVLAHIATAKYYDHLPLHRQLDIFEREGIHLSPSTVSNW
YP_001297710	PMPEGLSLEGATKLGEEVSEQYAVSPARFYVKRIIRPKYRLADGRIITAPMPVMAHPHSNASESVLAHIATAKYYDHLPLHRQLDIFEREGIHLSPSTVSNW
Contig 26741	MMAAAQRLEPIYNELRELVKDSYYVMADETPHPVLESDRPGALHRGYMWNFYLPRFHTPFFEYHKGRGSSGTDTLLAGQVRVVQSDGFAVY
YP_001297710	MMAAAQRLEPIYNELRELVKDSYYVMADETPHPVLESDRPGALHRGYMWNFYLPRFHTPFFEYHKGRGSSGIDTLLAGQVRVVQSDGFAVY
Contig 09258	ENSKPVPYDYFLTMRFWKEDLEHYLLYRECAQEDLEKTTWEPYRYSSYPGGTVALTQFINSHLKITPEMKATGKQGRVIYSFNVDIDGSMKDFRLVRGLDPLMD
YP_001299838	KNSKPVPYDYFLTMRFWKEDLEHYLLYRECAQEDLEKTTWEPYRYSSYPGGTVALTQFINSHLKITPEMKATGKQGRVIYSFNVDIDGSMKDFRLVRGLDPLMD
Contig 09258	AEALRVLQLVNEKWSTG
YP_001299838	AEALRVLQLVNEKWSTG
Contig 10937	FRSWPFPQQSFLVLLPLSQGCNFHKGIDGLCGEVIRHTGSCVSEQSCHIFPDRSRSRLHILYRCDDEYRLECRRLNRGSFLLKKEERKKDFLQISWNRLNELLT
YP_001297387	FRSSPFWSY---YLYP--QGCNFHKGIDGLCGEVIRHTGSCVSEQSCHIFPDRSRSRLHILYRCDDEYRLECRRLNRGSFLLKKEERKKDFLQISWNRLNELLT
Contig 10937	VKKYRKTVEK
YP_001297387	VKKYRKTVEK
Contig 25918	MLSALIRSRLRYPAMHLAAQPANQRSVQHGPLVLVSEPRKFHAPTIDRDRTVSRRSEPSSRATLMGEQPNPWDLLQPQDV
EDO52987	MLSALIQSRLRYPAMHLAAQPVNQRSVQHGPLVLVSEPRKFHAPTIDRDRTVSRRSEPSSRATLMGEQPNPWDLLQPQDV
Contig 25929	VFQPHLPVRLPCYDLAPVTSFTLGRSSRLRTSGTPGSHGLTGGVYKARERIHRAVADARLLANPA
EDO53852	MFQPHLPVRLPCYDLAPITSFTLGRSLRLRTSGTPGFHGLTGGVYKARERIHRAVADARLLANPA
Contig 25961	LAFHPYPQLIQKLFNAYWCGPPAGVTQPSTWPRVDHLVSRLPLPTIRPIQTRFRFGYVCRHT*PCRQRQLVGSLCKRHAVTH*RAPTACRRTVSGTISLFCSKCF
CAJ30045	MAFHPYPQIIPDFFNRRGFGPPVGVTPPSTCPWIDHSVSGLMHATRRPIQTRFRCAYTYRLKLAAYTNSLT-HYTKGTPSPFKRAPTACRHSVSGTVSLPLSGCF
Contig 25961	SPFPHGTGSLSVSREYLALPDGPGRFTQNSSCSALLR
CAJ30045	SPFPHGTSSLSVTEEYLGLEDGPPMFRQDFTCPALLK
Contig26526	MYQDERKLDFKPLGIAIKKAREAKGWTQEYLAQLVDLTPRSIMYIENRGQHPRLNKFYLITTLLDISVDQFFFPCNEDGDNNRRKQVDVLLNDMEEKELIVMEATAQGLKKA
EDP10382	MYQDERKLDFKPLGIAIKKAREAKGWTQEYLAQLVDLTPRSIMYIENRGQHPRLNKFYLITTLLDISVDQFFFPCNEDGDNNRRKQVDVLLNDMEEKELIVMEATAQGLKKA
Contig 27457	MEVNNKTAPVTGQQDQNTISLDLMNRMKLHGMAEAFRESLAGTTPQSMTADTFLSMLLAREWDYRSQAAIARLTKNAAFRYKAYIEQIDYATNRGLDRNQMERLATL
EDO51852	METNNLTAPIAVEKDRNTLTIELMNRMKLHGMAAAFTESLTSTMAETMTIDSFLHMLLAREWDYRANAAIQRLIRGAAFRYKACLEQIDYAIPRGLDRNQMERLASL
Contig 27457	DFVHKAQNLFITGSSGTGKSYLACALGHEACKRGFRTFYANAPkllgalkvakvkgTLEAELKKIERCQLLILDDLFIVPLDAKERPILLEIIEDRHERKSVIITSQ
EDO51852	EFIRKGQNLFITGSSGTGKSFLATAMGYEACKKGIRTYYANAPKLMGTLKVAKVKGTLESELKRIERSTLLILDDLFLVNLDAKERPILLDIIEDRHGRKSIIITSQ
Contig 27457	YPSSNWYDMVGDPTIA
EDO51852	LPTDNWYDAIGDPTVA
Contig 22050	MQQNIISNFFRPINTDIQIPD
YP_001298764	MQQNIISNFFRPINTDIQIPD
Contig 25094	PEGLPGRLIKLPLQAGNYDNFYSDGKKVWYASGRSTKVYDLTEQKEETVAEGAYMDVAANHRKALFFKGNNLYICDFPCTKASLEENVNLDDMIAPIDYSQEWA
YP_001298467	PEGLPGRLIKLPLQAGNYDNFYSDGKKVWYANGRSTKVYDLAKQKEEIVAEGAYMDVAANHRKALFFKGNNLYICDFPCTKASLEENINLSDMVAPIDYSQEWA
Contig 25094	QIFDETWRAFRDGFYLENMHGADWNAIKEKYAVLVPHAKTRLDLNYIIGEMIAELACGHAYVSPGEIKGPERIPMGLLG
YP_001298467	QIFDETWRAFRDGFYLENMHGADWNAIKEKYAVLVPHAKTRLDLNYIIGEMIAELACGHAYVNPGEIKGPERIPMGLLG
Contig 00991	IPIIPLKTFNIQNGSYVEWTGNIMNPQLNITATERVRASVGEDGKTSRIVGFDVGIALSQSLENLGLAFTLSAPEDASVQDQLNAMSVEERGKLAVTMLVTGMY
YP_001297716	IPIIPLKTFNIQNGSYVEWTGNIMNPQLNITAAERVRASVGEDGKTSRIVGFDVGIALSQSLENLGLAFTLSAPEDASVQDQLNAMSVEERGKLAVTMLVTGMY
Contig 00991	MAEGNSTGGFNVNNALNSFLQSEISNIAGKALDINVGMETVDDADSGGKRTDYNFQFAKRFWNNRFRI
YP_001297716	MAEGNSTGGFNVNNALNSFLQSEISNIAGKALDINVGMETVDDADSGGKRTDYNFQFAKRFWNNRFRI
Contig 26290	SSVGFHL--YWQVIPLFCNRDGFAQILAARK*LAWVSGRQTLAHFA-WLFLLNHDAKGTRVGLCCFLLMVFIAISSFPCGTVSLYCAGVVFSI
XP_454253	ASLGFGLINFFFAIPAFFMIDRFGRRFLLLNTFPWLAVFLLITGFSFWI-----DDTEKRIGVVSMGIYVFSAIYSFGCGVVPFVIAGEVFPL
Contig 25938	FPTEGTVRLFGTNYKENIHTLYSKVGSIIETPGFYSNLTGYENLQILAKLRGGVSKSGVEKALEVVGLHKEKRKVFSDYSLGMKQRLGIAAAIMHEPELLILDEPI
AAS78451 58	FPTDGTVRLFGTNYKENIHTLYSKVGSIIETPGFYSNLTGYENLQILAKLRGGVSKSGVEKALEVVGLHKEKRKVFSDYSLGMKQRLGIAAAIMHEPELLILDEPI
Contig 25938	NGLDPIGIVEIRSFLSELSHNHGITIFISSHVLSEIEQIADIIGVMHEGHLVEEVNISELHKRNRKYIQFDLSDSEIAGKILENHYH
AAS78451 188	NGLDPIGISEIRSFLSKLSHENGTTIFISSHVLSEIEQIADVIGVMHEGHLVEEVNISELHKRNRKYTEFDVSDGKIAAKILESSYH
Contig 25946	GSTRIKAVLIDQENKPIAQGSHSWENQLVDGLWTYSVEAIWHGLQDCYADLRSNVKKLYDTEIETLAAIGVSAMMHGYMAFNKEEEILVPFRTWRNTNTGPAAAAL
YP_001299600	GSTRIKAVLIDQENKPIAQGSHSWENQLVDGLWTYSVEAIWHGLQDCYADLRSNVKKLYDTEIETLAAIGVSAMMHGYMAFNKEEEILVPFRTWRNTNTGPAAAAL
Contig 25946	SELFVYNIPLRWSISHLYQAILDNEEHVSNIDYLTTLAGFIHWQITGQKVLGIGDASGMLPIDPATKNYSAEMI
YP_001299600	SELFVYNIPLRWSISHLYQAILDNEEHVSNIDYLTTLAGFIHWQITGQKVLGIGDASGMLPIDPATKNYSAEMI
Contig 26189	IGLIIIAIGSFCQSSSYVPIKKVKEWSWESFWLLQGVFAWLVFPLLGALLGIPQGSSLFDLWGTGGAPMSIFYGILWGVGGLTFGLSMRYLGVALGQSIALGTCA
YP_001297927	IGLIIIAIGSFCQSSSYVPIKKVKEWSWESFWLLQGVFAWLVFPLLGALLGIPQGSSLFDLWGTGGAPMSIFYGILWGVGGLTFGLSMRYLGVALGQSIALGTCA
Contig 26189	GFGTLFPAIFAGTNLFEGNGLILLLGVCITLSGIAIIGYAGSLRAKNMSEEEKRAAVKDFALTKGLLVALLAGVMSACFALGLDAGTPIKE
YP_001297927	GFGTLFPAIFAGTNLFEGNGLILLLGVCITLSGIAIIGYAGSLRAKNMSEEEKRAAVKDFALTKGLLVALLAGVMSACFALGLDAGTPIKE
Contig 27252	PSGKWAVHTFSNSETPPVIDMVSFPAHKSIRLITDNAKAKEQYKALGLQPKEFVKTRSGELELDAWMIKPVNFDPSKKYPVIIDVYGEPANATVQDVWSGGSLWHQ
YP_001297927	PSGKWAVHTFSNSETPPVIDMVSFPAHKSIRLITDNAKAKEQYKALGLQPKEFVKTRSGELELDAWMIKPVNFDPSKKYPVIIDVYGEPANATVQDVWSGGSLWHQ
Contig 27252	YLANLGYIIVSIENRGANAPRGRWRKCIYGEVGTFASEDQARGIQDLARQYSFIDTARIGITGWSGGGSQTLNS
YP_001297927	YLANLGYIIVSIENRGANAPRGRWRKCIYGEVGTFASEDQARGIQDLARQYSFIDTARIGITGWSGGGSQTLNS
Contig 03484	MGQYHRQGEQGHAKGRIRRTGLVCRLCIAGRSRGVRRKECHFRYQPVDMRYRIRKGLESPCMIRGLLSGDYWIFVGCCSAAFVLFFLGIRAGIS
YP_001298244	MGQYHRQGEQGHAKGRIRRTGLVCRLCIAGRSRGVRRKECHFRYQPVDMRYRIRKGLESPCMIRGLLSGDYWIFVGCCSAAFVLFFLGIRAGIS

## Discussion

The microbiome datasets presented herein represent the first assessment of the metabolic potential of the chicken cecum microbiome at the level functional gene content. As such, they represent a baseline for future studies and will be of great use in understanding the large, complex, and dynamic microbial community of the chicken cecum, the composition of which ultimately reflects the co-evolution/selection of microbes with their host and diet. It is clear that the composition and function the microbiome can be affected by various factors such as dietary ingredients, nutrient levels, environment, probiotic, and antibiotic treatments. Moreover, the gastrointestinal tract microbiome plays an important role in the growth and health of the host through its effects on gastrointestinal tract morphology, nutrition, pathogenesis of intestinal diseases, and immune responses. This comparative microbiome data provides a critical genetic context for understanding food safety, animal nutrition, animal health and well-being. Additionally, the combined pyrosequence approach and subsystems-based annotations available in the SEED database allowed us to gain an understanding of the metabolic potential of these microbiomes. Sequence information was recovered in a comparative context based on the ecology of the microbial communities that inhabit the chicken cecum, which in the future this will allow us to link metabolic potential to the identity of cecal microbes in their natural habitat.

Metagenomic analysis allows the relative abundances of all genes to be determined and used to generate a dataset for the assessment of the functional potential of each community [10], [48]–[53]. Our ability to assemble genes from primarily the *Bacteroidetes* suggests that this is an important phylum in the chicken cecum, similar to that observed in studies of the human fecal microbiome [46], [47]. We also note that while the community structure of the cecal microbiome from the *C. jejuni* challenged chicken has greater diversity and evenness with a distribution of more *Firmicutes* at the expense of the *Bacteroidetes* and other taxa. While this may suggest that the challenge affected the community structure in such a way as to diminish the levels of the dominant taxa, this may be the result of host variation in community structure. Even though the phylotype distribution was significantly different between the microbiome from a pathogen-free chicken compared with one that had been challenged by a single low-level inoculation with *C. jejuni*, the functional gene content of these two microbiomes was similar.

We have proposed from our metabiome analysis of 45 microbiomes and 42 viromes that the frequency of a gene encoding a particular metabolic function reflects its relative importance in an environment [12]. Interestingly, differences in functional and taxonomic evenness reported for microbial communities [51], [54] suggest that the frequency of a gene encoding a particular metabolic function reflects its relative importance in an environment. It also appears that like the human fecal microbiome, the chicken cecum microbiome contains an abundance of transferable elements including conjugative transposons, supporting the hypothesis that a driving force in microbiome composition and diversity in gastrointestinal tracts, or for that matter any environment of high microbial density, is horizontal gene transfer [47], [55]. Our observation that nearly 25% of the assembled contigs show similarity with mobile elements (transposases) is consistent with a microbiome evolution model that predicts that variation in gene content is mediated via horizontal gene transfer [56] which also controls gene distribution within the pan microbiome. This could be accelerated in this microbiome by the use of antibiotics, which is evidenced by the high proportion of antibiotic resistance genes detected in the chicken cecum microbiome. In this study we have primarily sampled the core microbiome of conserved, abundant genes within microbial metagenomes. This core microbiome is supplemented by a less abundant “variable microbiome” or “specialization genes” to provide those unique functions as and when required [57]. Our work supports the observation that the core genomes of widely distributed microbes remain essentially the same, with the main differences being prophage, pathogenicity, or ecological islands [58].

Our analysis allows us to extend the virulome concept of individual microbial groups into a metavirulome, which comprises all of the virulence components present in a microbiome, even if they are not directly involved in a overt disease in the primary host. This metavirulome can have a dramatic and direct evolutionary effect on mutualism, virulence acquisition and disease amongst commensal microbes. Dethelfsen et al. [59] recently reviewed this topic as it related to individual pathogens and the development of zoonotic pathogens, or those that are passed from animals to humans. Indeed, it appears that commensal microbes from domesticated species are the origin of many gastrointestinal diseases found in humans and other species [60]. For example, *C. jejuni* is a highly adapted to the avian gastrointestinal tract and is regarded as a commensal in the chicken. Nonetheless, *Campylobacter* species are recognized as important human pathogens, and are the most commonly identified bacterial cause of diarrheal illness in the world [61]. Thus, it would appear that the virulome of these bacteria are adapted to their primary host, and once transmission to a secondary host occurs the host immune system does not recognize the organism and colonization and disease can result. It would also appear that the metavirulome is a contributing factor in the development of low-virulence niches for the primary host-specific microbiome. Once these microbes are transmitted to different hosts that harbor distinctly different metavirulomes, the host environment could promote colonization of these zoonotic pathogens followed by a disease state in the new host. The entire microbiome must be considered in relationship to both ecological and evolutionary forces of the host and the microbial community, when considering the subtle differences in commensal and pathogenic microbes

## Materials and Methods

### Chicken Cecum Sampling

Chicks were obtained from a commercial hatchery (Murray McMurray, IA) and divided into two groups, A and B and housed in separate isolation buildings. Upon arrival, a cloacal swab from each bird was collected and plated on CEFEX media to ensure chicks were free of *Campylobacter*. All birds were fed a commercial chicken feed (Eagle milling, AZ) *ad libitum* for the duration of the study. Fourteen days post hatching, chicks in pen B were challenged via oral gavage with 1×10^5^ CFU *C. jejuni* NCTC11168. Chicks in pen A received only PBS and served as negative controls. Fourteen days post challenge, birds from each pen were euthanized and ceca collected for further analysis. Fresh cecal samples from two (*C. jejuni*-inoculated and *C. jejuni*-uninoculated) 28-day old chickens were analyzed. Cecal contents were collected using aseptic techniques. Samples were stored at −80°C until DNA extraction. These studies were approved by the Institutional Animal Care and Use Committee (IACUC) at the University of Arizona (Protocol#06-037), which assured adherence to humane and ethical principles, as outlined in the Animal Welfare Act, ILAR “Guide for Care and Use of Laboratory Animals,” and all other applicable public laws and local policies.

### DNA Extraction and Purification

Genomic DNA was extracted using a protocol similar to the extraction of high molecular weight DNA for rumen and fecal contents [62]. Deviation from this protocol included following the Qiagen DNA Stool Kit manufacturer's protocol (Qiagen, Valencia, CA) following the addition of 960 µl of ASL buffer to the samples. DNA purity and concentration was analyzed by spectrophotometric quantification and gel electrophoresis.

### Pyrosequencing and Sequence Analysis

The two cecal samples were subjected to a single pyrosequence run by 454 Life Sciences using a 454 Life Sciences Genome Sequencer GS20 and analyzed using the SEED Annotation Engine in MG-RAST (http://metagenomics.nmpdr.org; Version 1.2) [63]. The sequences were compared using the BLASTX algorithm with an expected cutoff of 1×10^−5^ [10]. The BLASTN algorithm (E<1×10^−5^ and a sequence length hit>50 nucleotides) was used to identify SSU rDNA genes from release 9.3.3 of the RDP database ([64]; http://rdp.cme.msu.edu/), and the European Ribosomal RNA database (http://www.psb.ugent.be/rRNA/index.html). RDP was used for robust Bacterial classification and the Europeans Ribosomal RNA database was used to classify Eukaryl and Archaeal sequences. The metagenomes used in this paper are freely available from the SEED platform and are being made accessible from CAMERA and the NCBI Short Read Archive. The NCBI genome project IDs used in this study are: 28597, and 28599.

### Diversity Indices

Shannon-Weiner, Simpson's lambda, and Pielou's evenness analyses for measuring species richness and evenness [43] for the SSU rDNA hits used the following equations; eq. 6.1 (Shannon-Weiner, or “Shannon's entropy”; p. 209), eq. 6.41 (Simpson's lambda, or “concentration;” p. 242), and eq. 6.44 (Pielou's evenness; p. 243) [43]. To estimate microbiome diversity, sets of random sequences from each microbiome and the maximum likelihood assemblage structure of assemblages was determined using mathematical rank-abundance models in PHAge Communities from Contig Spectra (PHACCS) ([44]; http://biome.sdsu.edu/phaccs). Random subsamples of the metagenomes were used instead of the totality of the whole metagenomes, because PHACCS analyses are more robust at low coverage.

### Statistics

To compare the distribution of taxonomic and functional groups between the two metagenomes a non-parametric Wilcoxon exact test was used. Non-parametric statistics were used because they have minimal assumption, except that the population distribution of the paired differences is assumed to be symmetric. The test takes into account the magnitude of the differences between two paired variables to identify whether significant differences exist. The data was normalized for sequencing efficiency by obtaining the percent distribution, prior to analysis. A separate test was conducted for each variable group.
